# Mapping disadvantage: identifying inequities in functional outcomes for prostate cancer survivors based on geography

**DOI:** 10.1186/s12885-022-09389-4

**Published:** 2022-03-17

**Authors:** Kendrick Koo, Nathan Papa, Melanie Evans, Michael Jefford, Maarten IJzerman, Victoria White, Sue M. Evans, Eli Ristevski, Jon Emery, Jeremy Millar

**Affiliations:** 1 Radiation Oncology, Alfred Health, Melbourne, Australia; 2 School of Public Health and Preventive Medicine, Monash University, Melbourne, Australia; 3 Department of Radiation Oncology, Peter MacCallum Cancer Centre, Victoria Melbourne, Australia; 4 Sir Peter MacCallum Department of Oncology, University of Melbourne, Victoria, Australia; 5 Department of Health Services Research, Peter MacCallum Cancer Centre, Victoria Melbourne, Australia; 6 Centre for Cancer Research, Cancer Health Services Research, University of Melbourne, Victoria, Australia; 7 School of Psychology, Deakin University, Victoria, Australia; 8 Cancer Council Victoria, Melbourne, Australia; 9 Monash Rural Health - Warragul, Monash University, Victoria, Australia

**Keywords:** Prostate cancer, Survivorship, Health policy, Geomapping, Functional outcomes, Quality of life, Socioeconomic disadvantage

## Abstract

**Background:**

Prostate cancer is the most common internal malignancy in Australian men, and although most patients have good survival outcomes, treatment toxicities can impair function, leading to diminished quality of life for prostate cancer survivors. Socioeconomic disadvantage and geographical remoteness have been shown to be related to worse oncologic outcomes, and it is expected that they would similarly influence functional outcomes in prostate cancer.

**Methods:**

Using data from the Victorian Prostate Cancer Outcomes Registry (*n* = 10,924), we investigated functional outcomes as measured by the Expanded Prostate Cancer Index Composite-26 (EPIC-26) following prostate cancer treatment, focusing on associations with socioeconomic status and geographical remoteness and controlling for clinicopathologic characteristics. A single composite score was developed from the five separate EPIC-26 domains for use in geo-mapping.

**Results:**

A total of 7690 patients had complete EPIC-26 data, allowing mapping hotspots of poor function using our composite score. These hotspots were observed to relate to areas of socioeconomic disadvantage. Significant heterogeneity in outcomes was seen in urban areas, with hotspots of good and poor function. Both socioeconomic disadvantage and geographical remoteness were found to predict for worse functional outcomes, although only the former is significant on multivariate analysis.

**Conclusions:**

Geo-mapping of functional outcomes in prostate cancer has the potential to guide health care service provision and planning. A nuanced policy approach is required so as not to miss disadvantaged patients who live in urban areas. We have demonstrated the potential of geo-mapping to visualise population-level outcomes, potentially allowing targeted interventions to address inequities in quality of care.

**Supplementary Information:**

The online version contains supplementary material available at 10.1186/s12885-022-09389-4.

## Background

Prostate cancer is the most common internal malignancy in Australian men with 19,508 men diagnosed in 2019, representing 25% of all male cancers [1]. Most patients present with early-stage disease, for which prostatectomy and radiation therapy are effective curative treatment modalities [2]. Hormone therapy plays a critical role in the neoadjuvant, adjuvant and salvage settings, whilst cytotoxic chemotherapy and a range of novel systemic therapies are used for men with metastatic and castrate-resistant disease [3].

Although most prostate cancer patients have good survival outcomes, functional outcomes in survivors are inconsistent and poor in some groups [4–6]. Prostate cancer survivors can experience life-long urinary, bowel and hormonal symptoms as well as loss of sexual function secondary to toxicities of treatment [7]. The impact of treatment toxicities on quality of life for prostate cancer survivors may be mitigated through early diagnosis and shared treatment decision making with clearer expectations from treatment [8]. Post-treatment, a range of interventions can improve men’s quality of life, including medical and surgical therapies to improve erectile function [9], reconstructive surgery for restoration of continence [10] as well as peer support and access to specialist nurses [11].

Despite recent progress in prostate cancer treatment and survivorship care, outcomes for patients remain unequal. There are clear geographical differences in survival outcomes, with a systematic review including six separate Australian studies suggesting higher disease-specific mortality in rural versus urban men [12]. It might be conjectured that a rural–urban divide also exists for functional outcome in prostate cancer survivors. This divide could result from a lack of specialist services being available outside major population centres, requiring men to have to travel to receive care [13].

Apart from the challenges associated with access to healthcare, there is Australian evidence that non-urban residency is inversely related to socioeconomic status, with lower educational attainment [14] and income [15]. Socioeconomic disadvantage has been associated with worse surgical outcomes [16] and is also associated with poorer cancer survival, with more advanced disease at presentation, and reduced access to treatment [17]. Specific to prostate cancer, a Swedish study found that disadvantaged patients presented with later stage disease and had a concomitant increase in disease-specific mortality [18]. The disparities in cancer mortality by socioeconomic disadvantage have been found to be worsening in Australia [19].

Socioeconomic disadvantage also leads to suboptimal survivorship outcomes. Whilst there have been no previous prostate cancer-specific studies in Australia, clinical follow-up and survivorship care for survivors of colorectal cancer in New South Wales were found to be deficient in socioeconomically disadvantaged patients, with increasing socioeconomic advantage associated with greater likelihood of guideline-concordant care [20]. It must however be highlighted that considerable socioeconomic disadvantage can also be found in urban areas and that non-urban areas are not homogenously disadvantaged [21].

Factors contributing to poor survival outcomes in cancer are intertwined: geographical locale and socioeconomic status are tightly interrelated and also influence disease stage at presentation and treatment modality, both of which are themselves linked [22]. It is reasonable to expect that these factors and their interrelationships would also influence functional outcome following prostate cancer treatment. If this is indeed found to be the case, it is imperative that these gaps are closed through the implementation of evidence-based policy and health service design. Interventions need to be concentrated on patients with the worst outcomes, and there is therefore a need to identify populations and geographic regions where symptom burden for prostate cancer survivors is particularly high.

In the current work, inequities in the functional outcomes of prostate cancer patients in Victoria – Australia’s second most populous state – are examined. We aim to identify the relative impact of geography and socioeconomic status on functional outcomes by providing a visual illustration through geographical mapping to facilitate policy discussion.

## Methods

### Overview

**Fig. 1:**
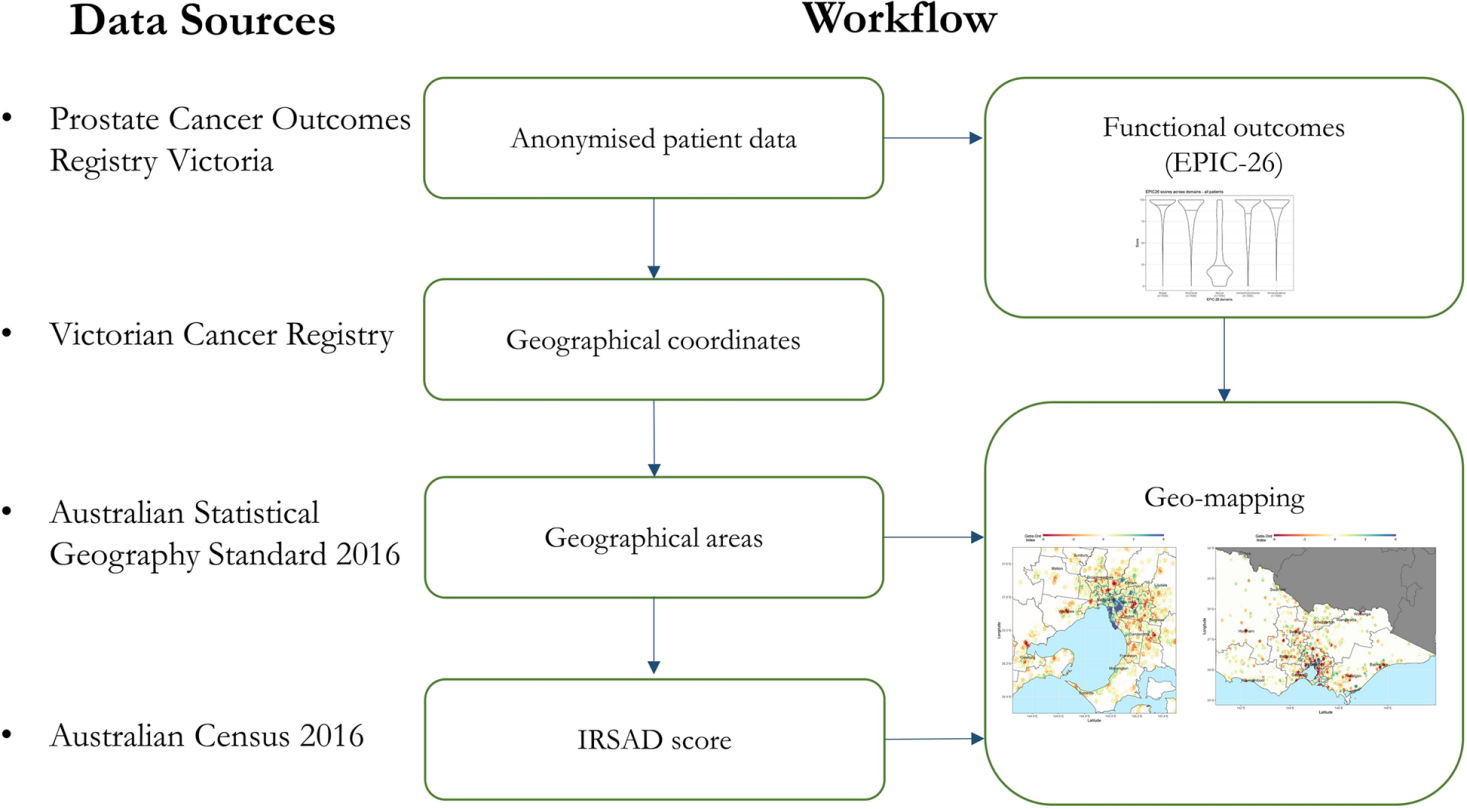
Schematic diagram illustrating data sources and data flow for geo-mapping of functional outcomes. IRSAD: Index of relative socioeconomic advantage and disadvantage; EPIC-26: Expanded Prostate Cancer Index Composite 26

This study was undertaken in Victoria, with a geographical area comparable to the United Kingdom [23] but approximately a tenth of the population [21, 24]. A summary diagram of data sources utilised and the overall geo-mapping workflow is presented in Fig. 1.

Anonymised patient data was retrieved from the Victorian Prostate Cancer Outcomes Registry (PCOR-Vic) which collects demographic, diagnostic, treatment and outcome data [25]. All patients enrolled into PCOR-Vic with a diagnosis of prostate cancer between September 2014 and December 2018 inclusive and a residential address in the state of Victoria with geographic coordinates (longitude and latitude) of patient residence at time of diagnosis obtained from linkage to the Victorian Cancer Registry.

Gleason grade, TNM stage and initial PSA levels were used to stratify patients into risk groups – “low”, “intermediate”, “high”, “nodal” and “metastatic” – in accordance with NCCN clinical practice guidelines [2]. Patients were grouped by treatment modality based upon first treatment received—surgery (open or robotic) or radiation therapy (external beam radiation therapy or brachytherapy) and active surveillance (no interventional treatment within the first 12 months) groups. Patients receiving androgen deprivation therapy but not having either surgery or radiation therapy were classified into the “ADT” group with remaining patients listed as “Other”.

### Quality of life metrics

Functional outcomes in PCOR-Vic are measured by the Expanded Prostate Cancer Index Composite 26 (EPIC-26) questionnaire, a validated tool assessing patient-reported quality of life in five separate domains—urinary incontinence, urinary irritative/obstructive, sexual, bowel and hormonal/vitality—for men with prostate cancer [26]. This questionnaire is administered 12 months post-treatment (or post-diagnosis for patients on observation). The questionnaire was initially administered by phone or sent out to patients by post but has been predominantly administered by email since April 2018, with a minority still completing the survey by phone or post.

Whilst a score of 100 in each of the five EPIC-26 domains indicates no decrement in function, the distribution of scores is inconsistent between domains, does not follow a well described statistical distribution and has strong ceiling effects, which all pose challenges in analysis [27]. Additionally, for the purposes of policy development, a single summary score would facilitate communication of findings to non-clinicians. We therefore propose a composite score, generated by first dividing the five domain-wise scores into quartiles and assigning a numerical value from 1 (worst) to 4 (best). For some EPIC-26 domains, the majority of patients had scores of 100, leading to identical thresholds for the top 2 quartiles. In these cases, the higher value is assigned. The sum of these values gives a derived score ranging from 5 (worst) to 20 (best), which follows a left-skewed Irwin-Hall distribution and following from the central limit theorem approximates a normal distribution.

### Geographic classification and socioeconomic status

Statistical Area (SA) geographic regions as defined by the Australian Statistical Geography Standard (2016) and published by the Australian Bureau of Statistics (ABS) were used [28]. To summarise this classification: SA1 is the smallest geographical unit for which census data is available and each have a population of 200—800 people; SA2 divisions represent amalgamations of socio-economically cohesive communities representing 3,000 – 25,000 people; SA3s are groups SA2s with similar regional characteristics and have populations between 30,000 – 130,000 people. Remoteness was classified according to the ABS remoteness structure, which separates the country into “Major cities”, “Inner regional”, “Outer regional”, “Remote” and “Very remote”. As no region in Victoria is classified as “Very remote”, and very few regions classified as “Remote”, these two categories have been combined with “Outer regional”.

Index of Relative Social Advantage and Disadvantage (IRSAD) scores are a validated measure of relative socio-economic advantage and disadvantage generated from the 2016 Australian Census data and provides a summary of economic and social conditions in a geographical area. Income and educational attainment are the primary inputs used to create this score. A low score indicates relatively greater disadvantage and lack of advantage, whilst a high score indicates a relative lack of disadvantage and greater advantage [29]. Patients were mapped to SA1 regions based on their geographical coordinates, with the IRSAD score for the respective SA1 division used to determine patient socioeconomic status.

### Regression analysis, geographic mapping and identification of hotspots

All analyses were performed in the R statistical programming environment. The *sf* [30] and *spdep* [31] R libraries were used for geospatial analysis.

For between-group comparisons, Fisher’s exact test was used for categorical variables and Kruskal–Wallis test for continuous variables. Univariate and multivariate regression was performed to investigate the contribution of IRSAD and geography to functional outcome. In regression analyses, NCCN risk groups were used instead of individual clinicopathologic factors due to the high collinearity between the individual factors.

There are drawbacks in using the raw EPIC-26 scores for geographic visualisation of overall functional status. Separate maps are needed for each functional domain and the inconsistent score distributions severely limits statistical analysis. We therefore used our EPIC-26 composite score to allow at-a-glance visualisation of functional outcomes across prostate cancer patients in Victoria, performing hotspot analysis to identify areas of low and high composite functional score. For this procedure, the map is tessellated with regular hexagons which are assigned the median score of patients mapped to each hexagon. Empty hexagons are assigned the median composite functional score of all patients. The hotspots are calculated from these hexagons using the Getis-Ord Gi* statistic [32] and visualised on the map.

## Results

### Overview

Data for a total of 10,924 patients were identified from the registry for the relevant time period who had a geocoded location of residence, of which 7690 (70% response rate) had complete data for all five EPIC-26 domains (Table 1). Only 14 patients self-identified as having Aboriginal or Torres Strait Islander ancestry.

**Table 1:** Clinicopathologic characteristics of analysed patients. Patient characteristics for 7690 prostate cancer patients identified from the PCOR-VIC registry with complete EPIC-26 data between September 2014 and December 2018 inclusive

	Complete (*n* = 7690) N (%)
Age – median (IQR)	67 (61–72)
Gleason Risk Group	
ISUP1	1964 (25.5)
ISUP2	2730 (35.5)
ISUP3	1399 (18.2)
ISUP4	705 (9.2)
ISUP5	892 (11.6)
T stage	
T1	3299 (42.9)
T2	1875 (24.4)
T3	508 (6.6)
T4	48 (0.6)
Not recorded	1960 (25.5)
N stage	
0	7262 (94.4)
1	312 (4.1)
Not recorded	116 (1.5)
M stage	
0	7276 (94.6)
1	346 (4.5)
Not recorded	68 (0.9)
PSA at diagnosis	6.8 (4.9–10.2)
NCCN risk group	
low	1639 (21.3)
intermediate	3737 (48.6)
high	1549 (20.1)
nodal	158 (2.1)
metastatic	346 (4.5)
Not classifiable	261 (3.4)
Treatment modality	
Prostatectomy	3985 (51.8)
WWAS	1831 (23.8)
Radiation therapy	1415 (18.4)
ADT	370 (4.8)
Other	89 (1.2)
Remoteness	
Major Cities	5461 (71)
Inner Regional	1750 (22.8)
Outer Regional	479 (6.2)
IRSAD – median (IQR)	1038 (974–1096)

*IQR* interquartile range, *WWAS* watchful waiting active surveillance, *IRSAD* index of relative socioeconomic advantage and disadvantage

Patients completing the questionnaire had a lower median age, lower risk disease, were more likely to have had a prostatectomy, live in regional areas and have a higher IRSAD score compared to those who did not complete the questionnaire (Supplementary Table 1).

For patients with complete data, IRSAD was evaluated against remoteness classification. Patients from major cities had the highest median IRSAD scores (indicating lower social disadvantage), followed by inner and outer regional areas, although there is significant heterogeneity within each remoteness category (Supplementary Fig. 1).

### Functional outcomes by composite score

The density distribution of the EPIC-26 composite score was visualised and confirmed to approximate a normal distribution (Supplementary Fig. 2). The quartile thresholds for each domain are tabulated in Supplementary Table 2.

**Fig. 2:**
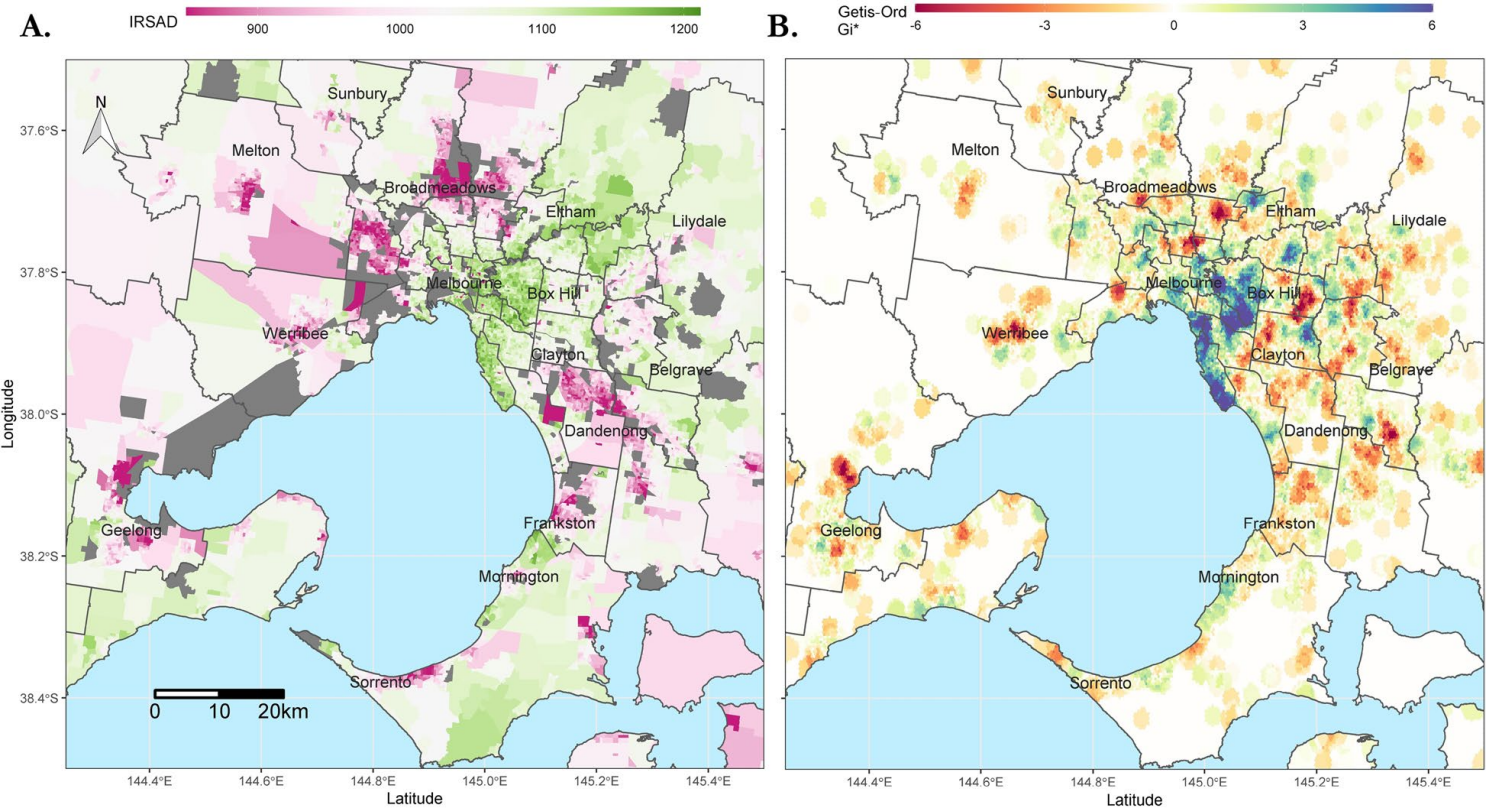
Mapping of IRSAD scores and hotspots of poor function for metropolitan Melbourne. Maps of metropolitan Melbourne overlaid by SA3 boundaries and coloured by: **A** IRSAD scores at SA1 resolution **B** Hotspots of poor function by composite score. Hotspots were identified using the Getis-Ord Gi* statistic, calculated across approximately 63,000 hexagons (0.135km^2^ each) for the map area. Colour bars representing values for each map are above the maps, with values to the left indicating lower IRSAD (brown) or a hotspot of poor function (red) and values to the right indicating higher IRSAD (teal) or a hotspot of good function (blue). The locations of major population centres are indicated on the map. Maps generated in R (version 3.5.1)

**Fig. 3:**
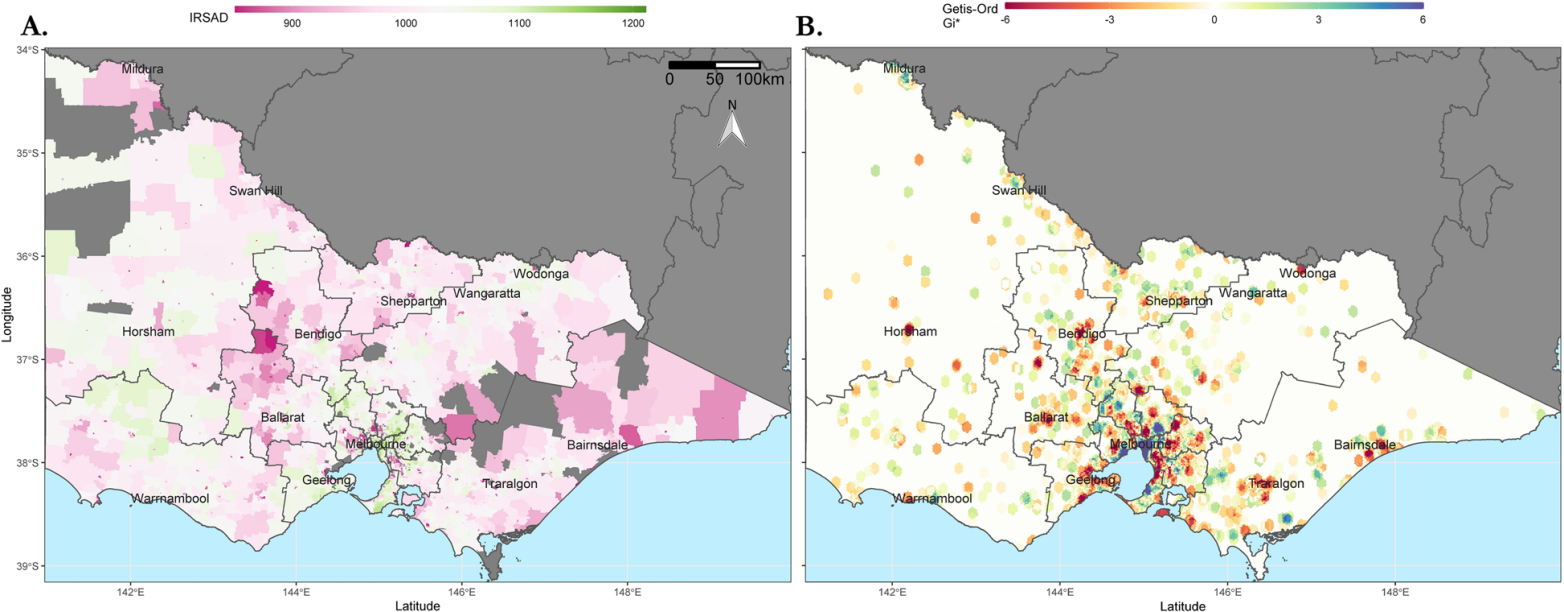
Mapping of IRSAD scores and hotspots of poor function for the state of Victoria. Maps of Victoria overlaid by SA3 boundaries and coloured by: **A** IRSAD scores at SA1 resolution **B** Hotspots of poor function by composite score. Hotspots were identified using the Getis-Ord Gi* statistic, calculated across approximately 118,000 hexagons (1.93km^2^ each) for the map area. Maps generated in R (version 3.5.1)

Hotspots of poor functional outcome were identified in the Melbourne metropolitan area (Fig. 2B) and when viewed side-by-side with a corresponding map of IRSAD scores (Fig. 2A), it is apparent that these areas of socioeconomic disadvantage contain hotspots of poor functional outcomes and areas of socioeconomic disadvantage contain “cold-spots” of good function. The heterogeneity of functional outcomes within the metropolitan area is striking, and there are also hotspots of poor function which fall in relatively socioeconomically advantaged areas. A similar map has been plotted for the entire state (Fig. 3) but the ability of this analysis to discern hotspots in sparsely populated regions is limited and these hotspots do not correspond as well to IRSAD score.

**Fig. 4:**
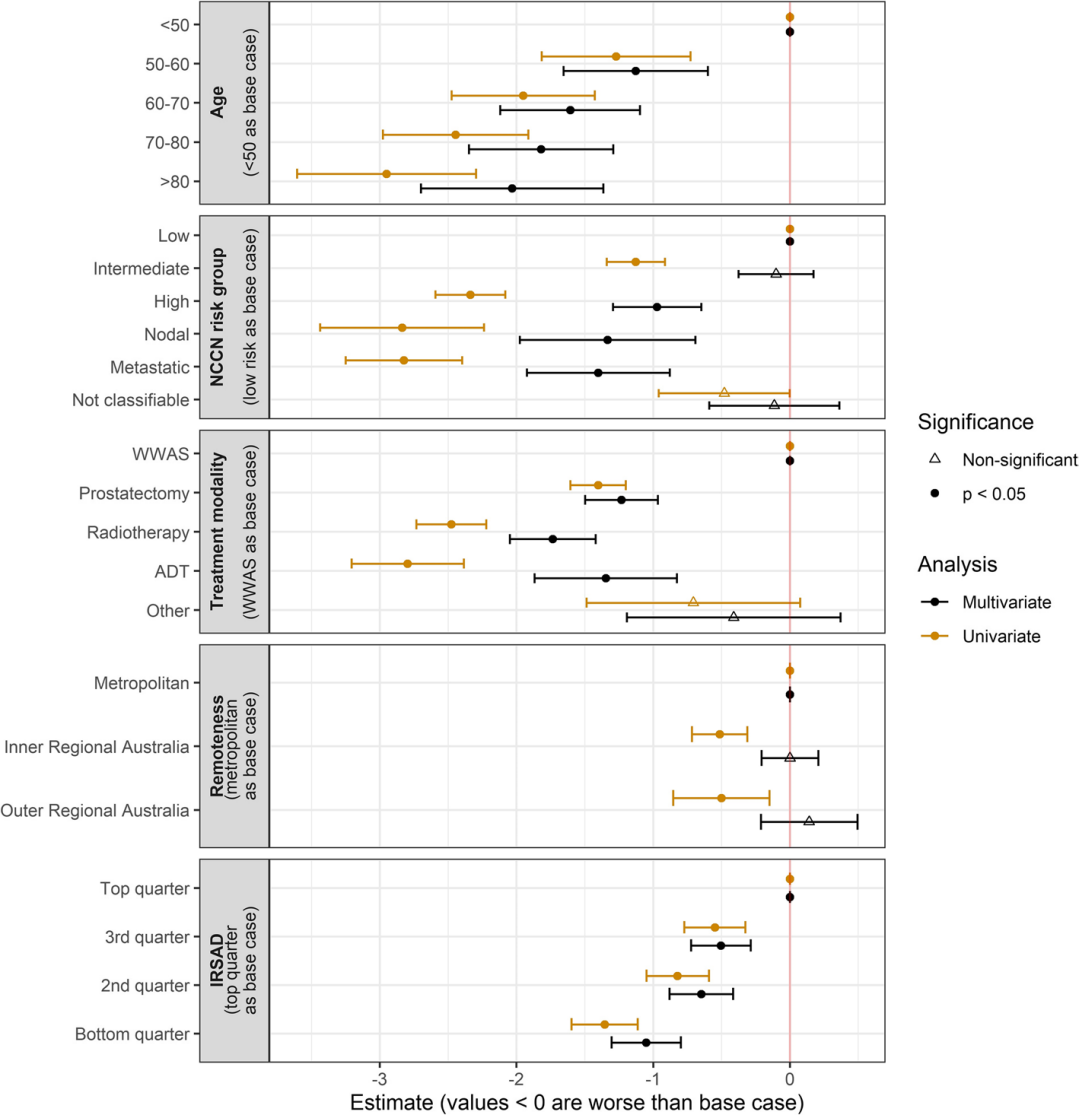
Multivariate analysis of clinicopathologic variables influencing functional outcome. Graphical illustration of linear regression coefficients, exploring changes to functional outcome by composite score for a range of clinicopathologic variables, coded into categories. The x-axis indicates the estimated coefficient i.e., an estimate of -1 represents a decrease of one point in the composite score. The raw data for this figure are available in Supplementary Table 3. WWAS: watchful waiting active surveillance; IRSAD: Index of Relative Social Advantage and Disadvantage

The predictors of functional outcome as measured by composite score were evaluated in a linear regression model (Fig. 4 and Supplementary Table 3). As expected, older age was associated with worse functional status, with increasing age resulting in a monotonic decrease in composite score (e.g., mean decrease in composite score of 1.1 points in 50–60-year-olds versus 2.0 in 70–80-year-olds compared to patients under 50 in the multivariate analysis). Having high risk disease (mean decrease of 1.0) or nodal (mean decrease of 1.3) or distant metastases (mean decrease of 1.4) was predictive for a lower composite score, but having intermediate risk disease did not independently predict for worse functional outcome. All treatment modalities were associated with worse functional outcome than active surveillance, with overlapping error bars for all modalities in the multivariate analysis.

Worse IRSAD, indicating greater socioeconomic disadvantage, and geographical remoteness both predict for lower composite score in the univariate analysis, with IRSAD in the bottom quarter of all patients being a particularly strong negative predictor of function (mean decrease of 1.4 points in composite score). On multivariate analysis however, only the relationship between low IRSAD to poor functional score remains significant. This relationship between low IRSAD and functional score is visualised in Supplementary Fig. 3.

### Functional outcomes by individual domains

**Fig. 5:**
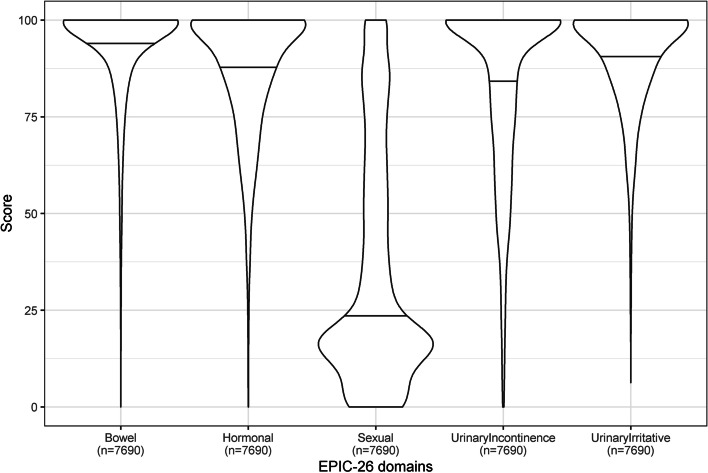
Scores for individual EPIC domain for all patients. Violin plot of each functional domain for the 7690 prostate cancer patients captured by the PCOR-VIC registry with complete EPIC-26 data. Horizontal lines indicate median score for each domain

To provide context for analysis of the composite score, we analysed functional outcomes as measured by each of the five individual EPIC-26 domains. When the domain-wise scores were visualised on violin plots, the considerable variation in the range and distribution of scores can be appreciated (Fig. 5), highlighting their unsuitability for geographic mapping. In particular, there are very strong ceiling effects in all except the “Sexual” domains, with a large proportion of patients having the maximum domain score of 100. By contrast, these ceiling effects are not apparent in the composite score (Supplementary Fig. 2).

Univariate and multivariate linear regression was performed to assess the contribution of disease characteristics, treatment modality, remoteness and socioeconomic status to scores in each EPIC-26 domain (Supplementary Tables 5, 6, 7 and 8).

IRSAD was the only variable found to be consistently significant on both univariate and multivariate analysis for almost every single EPIC-26 domain, with decreasing socioeconomic status predicting for worse functional outcome (e.g., mean decrease of 4.63 points in the Urinary Incontinence domain and 7.15 in the Sexual domain on multivariate analysis when comparing the top to the bottom quarter of IRSAD). The contribution of remoteness was much weaker, with regional residence ceasing to be significant for any domain in the multivariate analysis. Inner regional residence but not outer regional residence was significant in the univariate analysis for most domains, which may reflect diminished statistical power due to the small number of patients from outer regional areas.

Increasing age was a clear predictor for lower scores in the “Sexual” domain, and to a lesser extent for the “Urinary Incontinence” and “Urinary Irritative” domains, but had no apparent impact upon “Bowel” and “Hormonal” domain scores. High risk and nodal or distant metastatic disease predicted for worse outcomes in the “Sexual” and “Hormonal” domains, but had mixed results for the other domains.

The contrasting functional sequelae of different treatments became apparent when treatment modalities were compared to active surveillance. All modalities resulted in lower “Sexual” domain scores, albeit with impacts of varying magnitudes. Prostatectomy resulted in poorer “Urinary Incontinence” scores, whilst radiation therapy and ADT were both predictive for poor “Bowel” and “Hormonal” scores, in line with what is understood about these treatment modalities.

## Discussion

Through this analysis of PCOR-Vic, it is evident that socioeconomic status and remoteness influence functional outcomes following prostate cancer treatment. A novel composite score has also been developed, allowing geographical mapping and identification of regions of poor overall functional outcome.

Despite increasing socioeconomic disadvantage and remoteness both predicting for worse functional outcome following prostate cancer treatment, only socioeconomic disadvantage remains an independent predictor after controlling for confounding factors: older age [33] as well as higher risk disease and treatment [34] have previously been linked to poor functional outcomes and it was unsurprising to find these associated to functional outcomes in our data. Whilst remoteness does indeed predict worse functional outcome, our analysis suggests that this results from the interrelation between remoteness and socioeconomic disadvantage, the latter being the underlying driver of poor functional outcome. This finding is reinforced by the identification of hotspots of poor function within areas of socioeconomic disadvantage in urban areas on geo-mapping.

The association of functional outcome with socioeconomic status is not unexpected, but this finding is concerning. There has been recognition that there is an ethical imperative to ensure equity in cancer care [35] and there is an international effort to achieve this goal, with the American Society of Clinical Oncology formally committing to reaching cancer health equity [36]. These inequities in functional outcomes should be urgently addressed, although further research to identify the causative factors in each of the hotspots is required to guide policy development. Poor baseline functional status, later diagnosis as well as access to and quality of care are possible contributors to this inequity and tailoring of interventions to each hotspot is likely required. Advanced statistical techniques, including supervised and unsupervised machine learning methods, may aid in predicting poor functional outcomes and guide the interventions most likely to be of benefit, and will be the subject of further research.

The need for a single metric to evaluate functional outcomes has been demonstrated and we have proposed a composite EPIC-26 score to serve this purpose. Our proposed score has statistical properties allowing for comparison between cohorts of patients and geographical mapping, whilst retaining most of the major statistical associations with clinicopathologic characteristics. It is unavoidable that the nuance of individual domain scores has been lost: for example, variations in domain-specific outcomes between different treatment modalities are not discernible in the composite score. This loss of nuance is however offset by the benefit of avoiding ceiling effects in the composite score, which are well-recognised in the raw EPIC-26 instrument, particularly for the Bowel domain [27]. This does not correct for the ceiling effects in individual domains, but minimises bias and uncertainty when performing statistical tests [37]. This composite score will need to be validated in a wider cohort of patients and modifications may be required. In particular, we have weighted all functional domains equally in our work and fine tuning of weights for different domains may also be appropriate.

Geo-mapping and hotspot analysis has long been used in epidemiology, in particular to visualise the spatial distribution of infectious diseases [38]. However, we have identified no other published papers using this technique to assess quality of healthcare, and our work highlights the potential to extend this technique beyond its traditional role of visualisation of disease incidence. Refinements in technique and appropriate choice of metrics would allow geo-mapping to be applied to assess outcomes across other malignancies, but also all diseases more generally.

The primary limitation of this work is the use of an estimated measure of socioeconomic disadvantage based on place of residence, as socioeconomic data pertaining to individual patients is not captured in the registry, risking the ecological fallacy. However, the granularity of SA1 regions and broad use in Australia provide confidence in its suitability as a surrogate measure of socioeconomic disadvantage. Pre-treatment EPIC-26 scores were not captured, and it is therefore impossible to know if patients in areas of socioeconomic disadvantage had poorer function prior to treatment. Modifications to registry data collection protocols and to allow collection of pre-treatment functional scores will help to answer this question in the future.

As with any registry-based study, quality of registry data and adequate representation of the population are important caveats. The sampling fraction in this study is estimated to be 60% [39–43], and there remain geographical regions where registry data collection is deficient, particularly in selected regional areas in the North East of the state. Furthermore, not all patients completed the EPIC-26 questionnaire and there are systematic factors determining successful questionnaire completion, which may lead to bias in our observed associations. There is also low coverage of the Aboriginal and/or Torres Strait Islander population, which may reflect prevalence of prostate cancer in this community, but barriers to engagement with healthcare professionals may also play a role. The small numbers of Aboriginal and/or Torres Strait Islander included in these results precludes meaningful statistical analysis. Finally, other factors which may contribute to poor outcome including medical comorbidity and non-modifiable risk factors such as family history and ethnicity were not collected in the database and could not be included as covariates.

## Conclusion

We have demonstrated that remoteness and low IRSAD are both significant predictors of poor functional outcome following treatment for prostate cancer, with only the latter being an independent predictor. The utility of a composite EPIC-26 score for the purposes of geographic mapping has been demonstrated and we have identified hotspots of poor functional status in Victoria. From these results, we suggest that a more nuanced policy approach is required so as not to miss disadvantaged patients who live in metropolitan areas.

Finally, we suggest generalisation of our approach of mapping post-treatment functional outcomes as captured by a population-based registry for assessment of quality of care across diseases. Further development could result in an effective tool for managing health care service provision and planning, providing the capability to monitor population-level outcomes in real time as policy adjustments are made.

## Supplementary Information

**Additional file 1: **

## Data Availability

Data from the Australian Bureau of Statistics is freely available on their website (https://www.abs.gov.au/). The other datasets analysed during the current study are not publicly available, but applications can be made to PCOR-Vic and Cancer Council Victoria for data access.

## Abbreviations

ABS: Australian Bureau of Statistics (ABS)
ADT: Androgen Deprivation Therapy
ASGS: Australian Statistical Geography Standard
EPIC-26: Expanded Prostate Cancer Index Composite 26
IRSAD: Index of Relative Socioeconomic Advantage and Disadvantage
IQR: Interquartile range
NCCN: National Comprehensive Cancer Network
PSA: Prostate Specific Antigen
SA: Statistical Area
PCOR-Vic: Victorian Prostate Cancer Outcomes Registry
WWAS: Watchful waiting active surveillance
