# Supplementary material for: Mapping disadvantage: identifying inequities in functional outcomes for prostate cancer survivors based on geography
Source: BMC Cancer. 2022 Mar 17;22:283. doi: 10.1186/s12885-022-09389-4 (PMC8928643; doi:10.1186/s12885-022-09389-4)
Supplement: Supplementary file 1 — Additional file 1: [file 12885_2022_9389_MOESM1_ESM.docx]

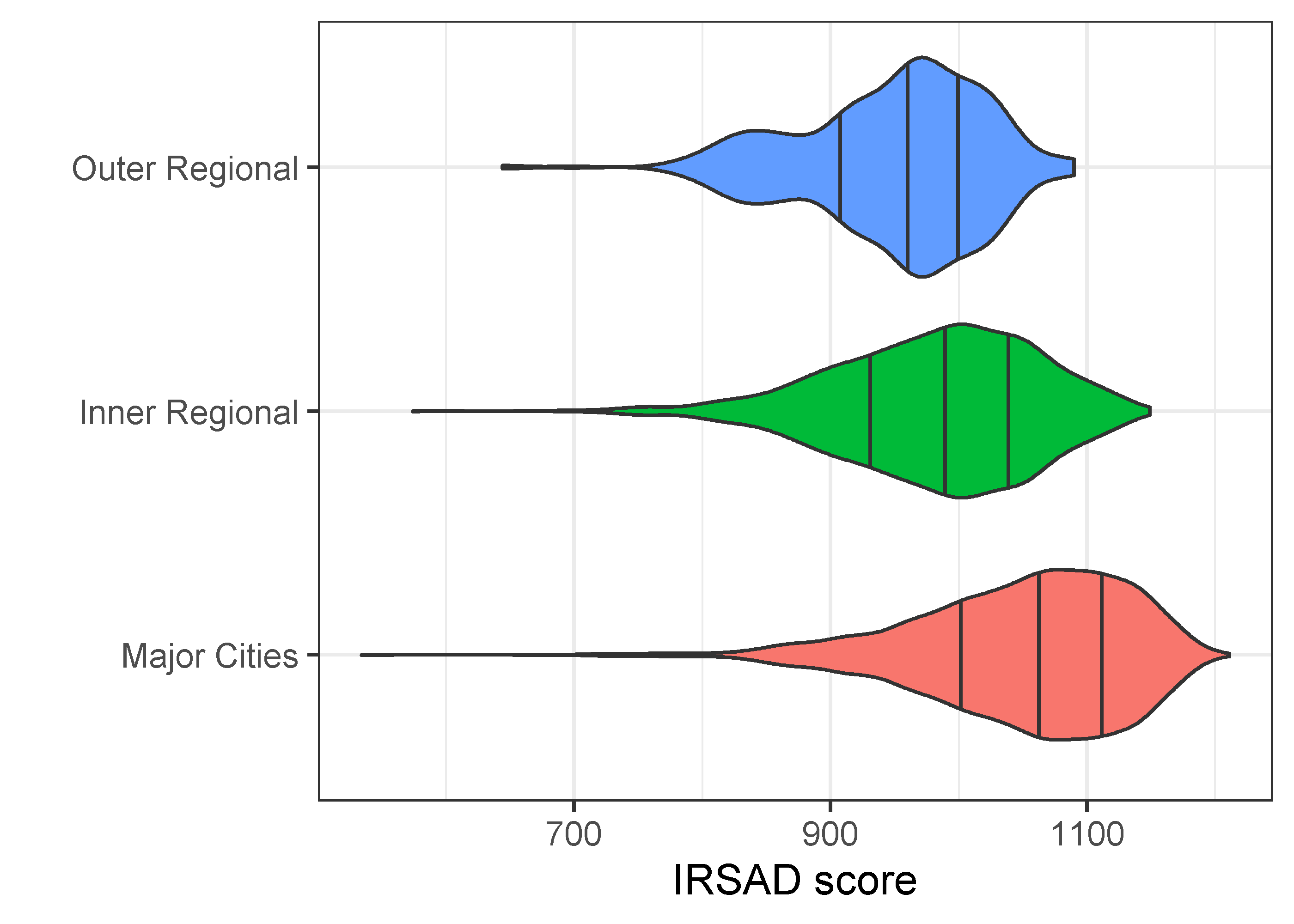


Supplementary Figure 1. Violin plots showing IRSAD score distribution for prostate cancer patients in Victoria, stratified by remoteness classification. Vertical lines divide distributions into quartiles.


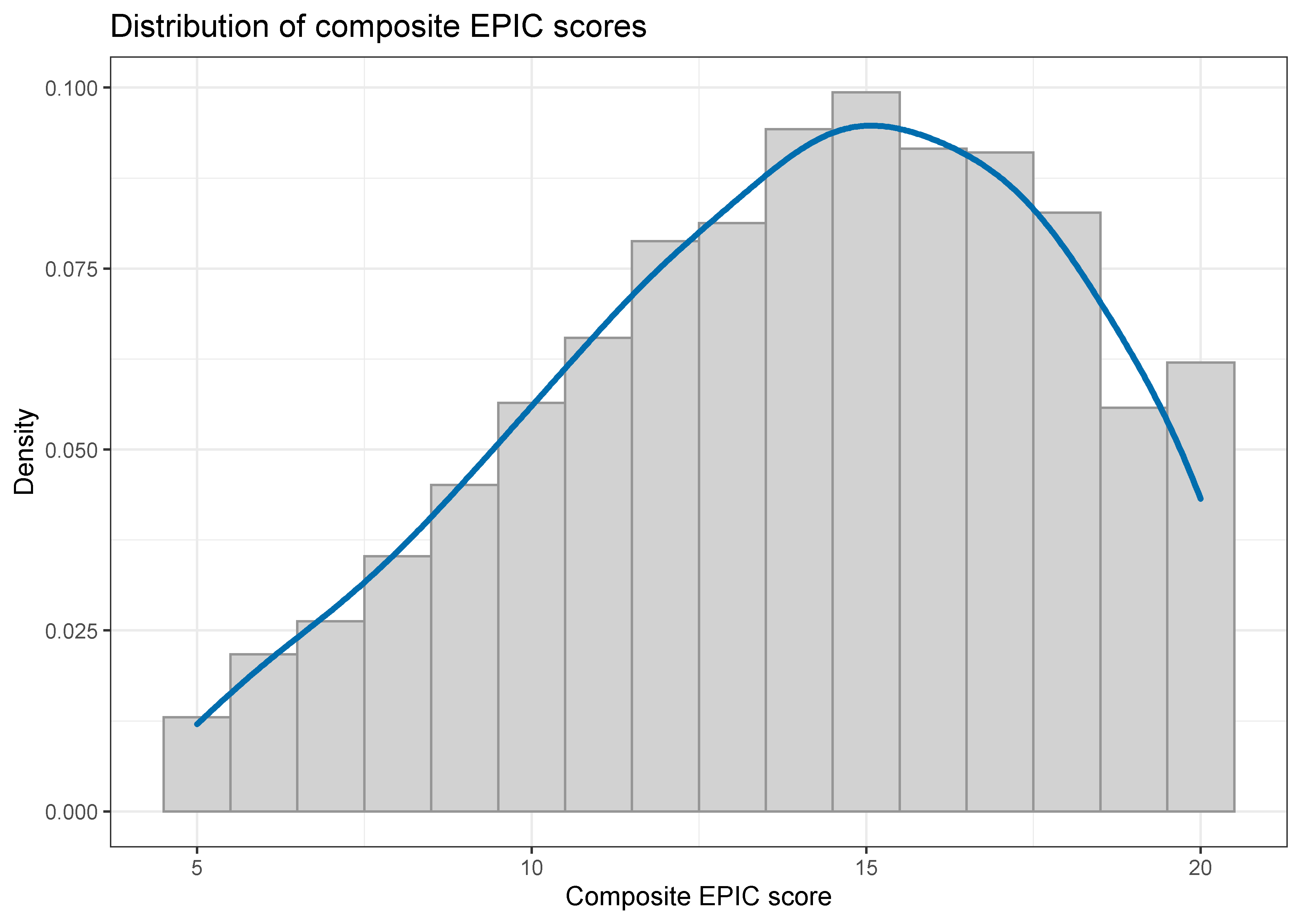


Supplementary Figure 2. Distribution of composite scores


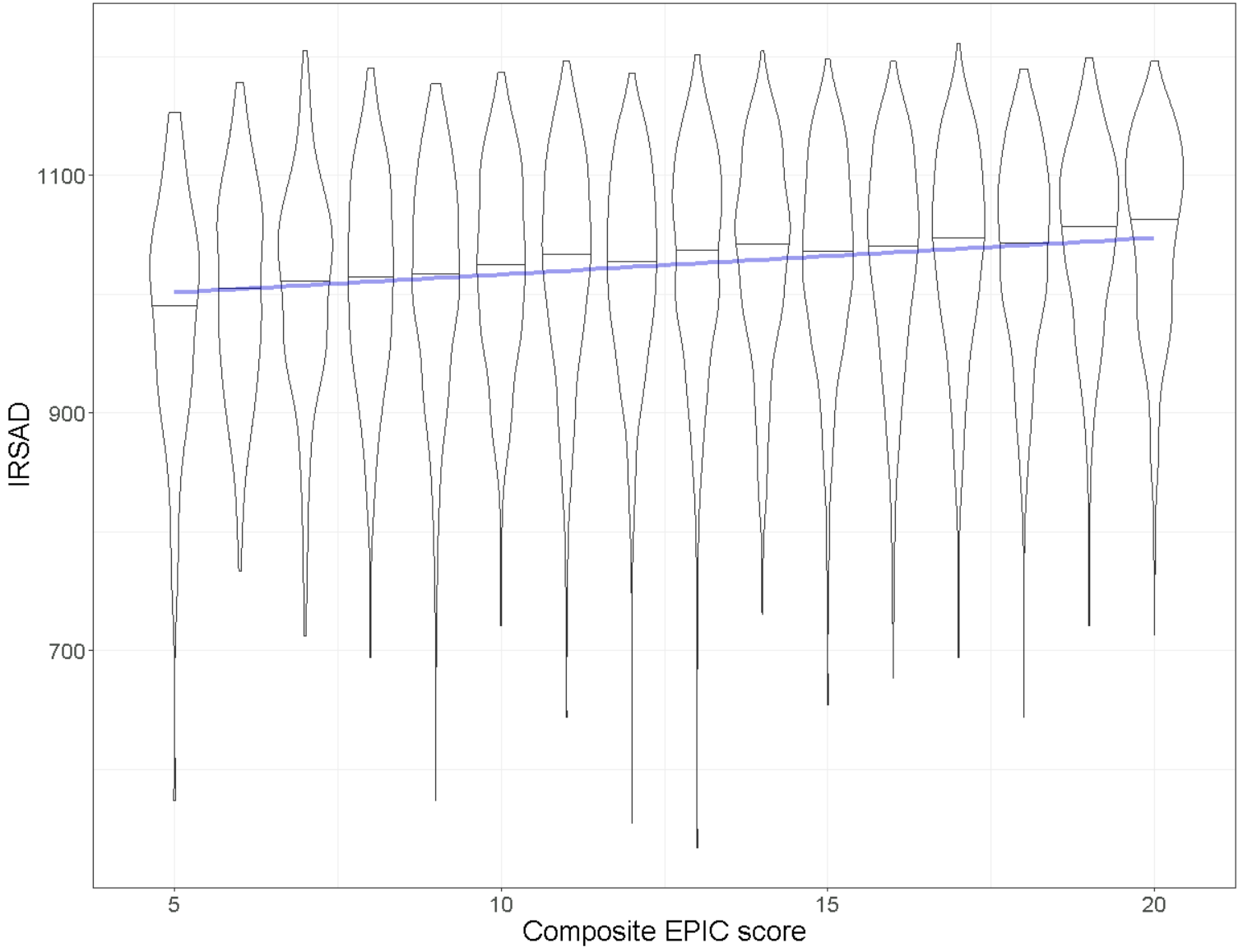


Supplementary Figure 3. Relationship of composite scores to socioeconomic index. A univariate linear regression model (Supplementary Table 3) is represented by the blue line.

Supplementary Table 1. Comparison of clinicopathologic variables between patients completing all sections of the EPIC-26 questionnaire and patients who did not complete the questionnaire, for the study period.

|  | Complete (n = 7690)  N (%) | Incomplete (n = 3234)  N (%) | p |
| --- | --- | --- | --- |
| Age – median (IQR) | 67 (61-72) | 69 (63-76) | <0.001 |
| Gleason Risk Group |  |  |  |
| - Grade group 1 | 1964 (25.5) | 961 (29.7) | <0.001 |
| - Grade group 2 | 2730 (35.5) | 836 (25.9) |  |
| - Grade group 3 | 1399 (18.2) | 482 (14.9) |  |
| - Grade group 4 | 705 (9.2) | 310 (9.6) |  |
| - Grade group 5 | 892 (11.6) | 645 (19.9) |  |
| T stage |  |  |  |
| - T1 | 3299 (42.9) | 1133 (35) | <0.001 |
| - T2 | 1875 (24.4) | 668 (20.7) |  |
| - T3 | 508 (6.6) | 333 (10.3) |  |
| - T4 | 48 (0.6) | 73 (2.3) |  |
| - Not recorded | 1960 (25.5) | 1027 (31.8) |  |
| N stage |  |  |  |
| - N0 | 7262 (94.4) | 2912 (90) | <0.001 |
| - N+ | 312 (4.1) | 232 (7.2) |  |
| - Not recorded | 116 (1.5) | 90 (2.8) |  |
| M stage |  |  |  |
| - M0 | 7276 (94.6) | 2866 (88.6) | <0.001 |
| - M1 | 346 (4.5) | 329 (10.2) |  |
| - Not recorded | 68 (0.9) | 39 (1.2) |  |
| Initial PSA – median (IQR) | 6.8 (4.9-10.2) | 8 (5.3-14.1) | <0.001 |
| NCCN risk group |  |  |  |
| - low | 1639 (21.3) | 787 (24.3) | <0.001 |
| - intermediate | 3737 (48.6) | 1081 (33.4) |  |
| - high | 1549 (20.1) | 814 (25.2) |  |
| - nodal | 158 (2.1) | 94 (2.9) |  |
| - metastatic | 346 (4.5) | 329 (10.2) |  |
| - Not classifiable | 261 (3.4) | 129 (4) |  |
| Treatment modality |  |  |  |
| - Prostatectomy | 3985 (51.8) | 952 (29.4) | <0.001 |
| - WWAS | 1831 (23.8) | 1033 (31.9) |  |
| - Radiotherapy | 1415 (18.4) | 666 (20.6) |  |
| - ADT | 370 (4.8) | 378 (11.7) |  |
| - Other | 89 (1.2) | 205 (6.3) |  |
| Remoteness |  |  |  |
| - Major Cities of Australia | 5461 (71.0) | 2401 (74.2) | 0.003 |
| - Inner Regional Australia | 1750 (22.8) | 660 (20.4) |  |
| - Outer Regional Australia | 479 (6.2) | 173 (5.3) |  |
| IRSAD – median (IQR) | 1038 (974-1096) | 1021 (949-1081) | <0.001 |

IQR: interquartile range; WWAS: watchful waiting active surveillance; IRSAD: index of relative socioeconomic advantage and disadvantage;

|  | Minimum score required for each quartile | | | |
| --- | --- | --- | --- | --- |
| **Assigned Score** | **1** | **2** | **3** | **4** |
| Urinary Irritative | 6.25 | 87.5 | 93.7 | 100 |
| Urinary Incontinence | 0 | 73 | 93.7 | 100 |
| Sexual | 0 | 12.5 | 20.8 | 58.3 |
| Bowel | 0 | 91.6 | 100 | 100 |
| Hormonal | 0 | 80 | 95 | 100 |

Supplementary Table 2. Score thresholds for each quartile for the five EPIC-26 domains.

Supplementary Table 3. Univariate and multivariate linear regression, exploring contributions of disease characteristics, treatment modality, remoteness and socioeconomic status to composite EPIC scores. * indicates p < 0.05

|  | Univariate | | | | Multivariate | | | |
| --- | --- | --- | --- | --- | --- | --- | --- | --- |
|  | Estimate | SE | t | p | Estimate | SE | t | p |
| Age in years (<50 as base case) |  |  |  |  |  |  |  |  |
| - 50-60 | -1.27 | 0.28 | -4.59 | <0.001* | -1.13 | 0.27 | -4.19 | <0.001* |
| - 60-70 | -1.95 | 0.27 | -7.30 | <0.001* | -1.61 | 0.26 | -6.16 | <0.001* |
| - 70-80 | -2.45 | 0.27 | -9.00 | <0.001* | -1.82 | 0.27 | -6.75 | <0.001* |
| - >80 | -2.95 | 0.33 | -8.84 | <0.001* | -2.03 | 0.34 | -5.96 | <0.001* |
| NCCN risk group  (low risk as base case) |  |  |  |  |  |  |  |  |
| - Intermediate | -1.13 | 0.11 | -10.4 | <0.001* | -0.10 | 0.14 | -0.72 | 0.47 |
| - High | -2.34 | 0.13 | -17.96 | <0.001* | -0.97 | 0.17 | -5.90 | <0.001* |
| - Nodal | -2.84 | 0.31 | -9.27 | <0.001* | -1.33 | 0.33 | -4.07 | <0.001* |
| - Metastatic | -2.82 | 0.22 | -13.00 | <0.001* | -1.40 | 0.27 | -5.25 | <0.001* |
| - Not classifiable | -0.48 | 0.25 | -1.96 | 0.05* | -0.11 | 0.24 | -0.47 | 0.64 |
| Treatment Modality  (Active surveillance as base case) |  |  |  |  |  |  |  |  |
| - Prostatectomy | -1.40 | 0.10 | -13.54 | <0.001* | -1.23 | 0.14 | -9.08 | <0.001* |
| - Radiotherapy | -2.48 | 0.13 | -19.06 | <0.001* | -1.74 | 0.16 | -10.87 | <0.001* |
| - ADT | -2.80 | 0.21 | -13.36 | <0.001* | -1.35 | 0.27 | -5.07 | <0.001* |
| - Other | -0.71 | 0.40 | -1.77 | 0.08 | -0.41 | 0.40 | -1.03 | 0.30 |
| Remoteness classification  (Major cities as base case) |  |  |  |  |  |  |  |  |
| - Inner Regional | -0.51 | 0.10 | -4.96 | <0.001* | 0.001 | 0.11 | 0.01 | 0.99 |
| - Outer Regional | -0.50 | 0.18 | -2.79 | 0.005* | 0.14 | 0.18 | 0.78 | 0.43 |
| IRSAD in quarters  (top quarter as base case) |  |  |  |  |  |  |  |  |
| - 3^rd^ quarter | -0.55 | 0.11 | -4.82 | <0.001* | -0.50 | 0.11 | -4.53 | <0.001* |
| - 2^nd^ quarter | -0.82 | 0.12 | -7.05 | <0.001* | -0.65 | 0.12 | -5.48 | <0.001* |
| - Bottom quarter | -1.36 | 0.12 | -10.97 | <0.001* | -1.05 | 0.13 | -8.13 | <0.001* |

Supplementary Table 4. Univariate and multivariate linear regression, exploring contributions of disease characteristics, treatment modality, remoteness and socioeconomic status to scores in the EPIC-26 “Urinary Incontinence” domain. * indicates p < 0.05

|  | Univariate | | | | Multivariate | | | |
| --- | --- | --- | --- | --- | --- | --- | --- | --- |
|  | Estimate | SE | t | p | Estimate | SE | t | p |
| Age in years (<50 as base case) |  |  |  |  |  |  |  |  |
| - 50-60 | -3.50 | 1.67 | -2.10 | 0.036* | -4.10 | 1.60 | -2.56 | 0.010* |
| - 60-70 | -6.37 | 1.61 | -3.96 | <0.001* | -6.77 | 1.55 | -4.36 | <0.001* |
| - 70-80 | -6.65 | 1.64 | -4.07 | <0.001* | -8.94 | 1.60 | -5.57 | <0.001* |
| - >80 | -3.45 | 2.01 | -1.72 | 0.086 | -10.02 | 2.03 | -4.94 | <0.001* |
| NCCN risk group  (low risk as base case) |  |  |  |  |  |  |  |  |
| - Intermediate | -7.84 | 0.66 | -11.90 | <0.001* | -0.10 | 0.83 | -0.12 | 0.903 |
| - High | -10.06 | 0.79 | -12.76 | <0.001* | -3.49 | 0.98 | -3.56 | <0.001* |
| - Nodal | -4.21 | 1.85 | -2.27 | 0.023* | -1.84 | 1.95 | -0.95 | 0.344 |
| - Metastatic | -4.61 | 1.32 | -3.51 | <0.001* | -3.91 | 1.59 | -2.47 | 0.014* |
| - Not classifiable | -2.09 | 1.48 | -1.41 | 0.158 | -0.16 | 1.44 | -0.11 | 0.909 |
| Treatment Modality  (Active surveillance as base case) |  |  |  |  |  |  |  |  |
| - Prostatectomy | -14.00 | 0.61 | -22.82 | <0.001* | -13.64 | 0.81 | -16.91 | <0.001* |
| - Radiotherapy | -3.64 | 0.77 | -4.74 | <0.001* | -0.61 | 0.95 | -0.64 | 0.520 |
| - ADT | -3.79 | 1.24 | -3.07 | 0.002* | 1.20 | 1.58 | 0.76 | 0.449 |
| - Other | -5.89 | 2.35 | -2.50 | 0.012* | -4.87 | 2.37 | -2.05 | 0.040* |
| Remoteness classification  (Major cities as base case) |  |  |  |  |  |  |  |  |
| - Inner Regional | -1.38 | 0.62 | -2.24 | 0.025* | 0.17 | 0.63 | 0.27 | 0.790 |
| - Outer Regional | -1.48 | 1.07 | -1.38 | 0.168 | -0.10 | 1.07 | -0.09 | 0.929 |
| IRSAD in quarters  (top quarter as base case) |  |  |  |  |  |  |  |  |
| - 3^rd^ quarter | -2.06 | 0.69 | -3.00 | 0.003* | -2.03 | 0.66 | -3.07 | 0.002* |
| - 2^nd^ quarter | -2.20 | 0.70 | -3.14 | 0.002* | -2.48 | 0.70 | -3.53 | <0.001* |
| - Bottom quarter | -3.63 | 0.74 | -4.89 | <0.001* | -4.63 | 0.77 | -6.01 | <0.001* |

Supplementary Table 5. Univariate and multivariate linear regression, exploring contributions of disease characteristics, treatment modality, remoteness and socioeconomic status to scores in the EPIC-26 “Urinary Irritative” domain. * indicates p < 0.05

|  | Univariate | | | | Multivariate | | | |
| --- | --- | --- | --- | --- | --- | --- | --- | --- |
|  | Estimate | SE | t | p | Estimate | SE | t | p |
| Age in years (<50 as base case) |  |  |  |  |  |  |  |  |
| - 50-60 | -2.55 | 0.96 | -2.65 | 0.008* | -1.98 | 0.95 | -2.08 | 0.037* |
| - 60-70 | -2.87 | 0.93 | -3.10 | 0.002* | -2.11 | 0.92 | -2.28 | 0.022* |
| - 70-80 | -3.33 | 0.94 | -3.54 | <0.001* | -1.62 | 0.95 | -1.70 | 0.089 |
| - >80 | -4.85 | 1.16 | -4.19 | <0.001* | -1.25 | 1.21 | -1.04 | 0.301 |
| NCCN risk group  (low risk as base case) |  |  |  |  |  |  |  |  |
| - Intermediate | 1.80 | 0.38 | 4.70 | <0.001* | -0.66 | 0.49 | -1.34 | 0.181 |
| - High | 0.57 | 0.46 | 1.25 | 0.212 | -1.20 | 0.58 | -2.06 | 0.039* |
| - Nodal | -2.52 | 1.08 | -2.34 | 0.019* | -3.19 | 1.16 | -2.75 | 0.006* |
| - Metastatic | -1.32 | 0.77 | -1.72 | 0.085 | -1.67 | 0.94 | -1.77 | 0.076 |
| - Not classifiable | -1.04 | 0.86 | -1.20 | 0.229 | -1.21 | 0.86 | -1.41 | 0.159 |
| Treatment Modality  (Active surveillance as base case) |  |  |  |  |  |  |  |  |
| - Prostatectomy | 4.35 | 0.36 | 12.06 | <0.001* | 4.81 | 0.48 | 10.04 | <0.001* |
| - Radiotherapy | -0.25 | 0.45 | -0.54 | 0.586 | 0.53 | 0.56 | 0.94 | 0.349 |
| - ADT | -0.08 | 0.73 | -0.11 | 0.911 | 1.28 | 0.94 | 1.36 | 0.173 |
| - Other | 2.57 | 1.39 | 1.85 | 0.064 | 3.05 | 1.41 | 2.16 | 0.030* |
| Remoteness classification  (Major cities as base case) |  |  |  |  |  |  |  |  |
| - Inner Regional | -0.49 | 0.36 | -1.37 | 0.172 | 0.14 | 0.38 | 0.37 | 0.709 |
| - Outer Regional | -0.20 | 0.62 | -0.32 | 0.748 | 0.82 | 0.64 | 1.29 | 0.198 |
| IRSAD in quarters  (top quarter as base case) |  |  |  |  |  |  |  |  |
| - 3^rd^ quarter | -1.01 | 0.39 | -2.55 | 0.011* | -1.04 | 0.39 | -2.64 | 0.008* |
| - 2^nd^ quarter | -0.93 | 0.40 | -2.31 | 0.021* | -0.76 | 0.42 | -1.82 | 0.069 |
| - Bottom quarter | -2.39 | 0.43 | -5.59 | <0.001* | -1.88 | 0.46 | -4.11 | <0.001* |

Supplementary Table 6. Univariate and multivariate linear regression, exploring contributions of disease characteristics, treatment modality, remoteness and socioeconomic status to scores in the EPIC-26 “Sexual” domain. * indicates p < 0.05

|  | Univariate | | | | Multivariate | | | |
| --- | --- | --- | --- | --- | --- | --- | --- | --- |
|  | Estimate | SE | t | p | Estimate | SE | t | p |
| Age in years (<50 as base case) |  |  |  |  |  |  |  |  |
| - 50-60 | -15.08 | 2.15 | -7.00 | <0.001* | -14.93 | 1.88 | -7.92 | <0.001* |
| - 60-70 | -28.39 | 2.08 | -13.68 | <0.001* | -26.08 | 1.83 | -14.27 | <0.001* |
| - 70-80 | -37.26 | 2.11 | -17.65 | <0.001* | -34.42 | 1.89 | -18.25 | <0.001* |
| - >80 | -41.64 | 2.59 | -16.06 | <0.001* | -40.58 | 2.39 | -17.01 | <0.001* |
| NCCN risk group  (low risk as base case) |  |  |  |  |  |  |  |  |
| - Intermediate | -23.42 | 0.82 | -28.57 | <0.001* | -1.79 | 0.98 | -1.83 | 0.068 |
| - High | -35.90 | 0.98 | -36.62 | <0.001* | -10.97 | 1.15 | -9.51 | <0.001* |
| - Nodal | -35.20 | 2.31 | -15.27 | <0.001* | -11.38 | 2.29 | -4.97 | <0.001* |
| - Metastatic | -37.03 | 1.64 | -22.62 | <0.001* | -14.92 | 1.87 | -7.99 | <0.001* |
| - Not classifiable | -9.96 | 1.84 | -5.40 | <0.001* | -2.96 | 1.70 | -1.74 | 0.081 |
| Treatment Modality  (Active surveillance as base case) |  |  |  |  |  |  |  |  |
| - Prostatectomy | -31.41 | 0.77 | -41.00 | <0.001* | -29.94 | 0.95 | -31.56 | <0.001* |
| - Radiotherapy | -31.46 | 0.96 | -32.72 | <0.001* | -21.05 | 1.12 | -18.85 | <0.001* |
| - ADT | -40.44 | 1.55 | -26.12 | <0.001* | -22.09 | 1.86 | -11.89 | <0.001* |
| - Other | -21.41 | 2.95 | -7.26 | <0.001* | -17.89 | 2.79 | -6.41 | <0.001* |
| Remoteness classification  (Major cities as base case) |  |  |  |  |  |  |  |  |
| - Inner Regional | -4.77 | 0.83 | -5.72 | <0.001* | 0.46 | 0.74 | 0.62 | 0.535 |
| - Outer Regional | -4.60 | 1.45 | -3.18 | 0.001* | 0.82 | 1.26 | 0.65 | 0.515 |
| IRSAD in quarters  (top quarter as base case) |  |  |  |  |  |  |  |  |
| - 3^rd^ quarter | -3.73 | 0.92 | -4.06 | <0.001* | -3.33 | 0.78 | -4.27 | <0.001* |
| - 2^nd^ quarter | -7.45 | 0.94 | -7.93 | <0.001* | -5.73 | 0.83 | -6.92 | <0.001* |
| - Bottom quarter | -9.69 | 1.00 | -9.73 | <0.001* | -7.15 | 0.91 | -7.90 | <0.001* |

Supplementary Table 7. Univariate and multivariate linear regression, exploring contributions of disease characteristics, treatment modality, remoteness and socioeconomic status to scores in the EPIC-26 “Bowel” domain. * indicates p < 0.05

|  | Univariate | | | | Multivariate | | | |
| --- | --- | --- | --- | --- | --- | --- | --- | --- |
|  | Estimate | SE | t | p | Estimate | SE | t | p |
| Age in years (<50 as base case) |  |  |  |  |  |  |  |  |
| - 50-60 | -0.09 | 0.89 | -0.10 | 0.918 | 0.41 | 0.88 | 0.47 | 0.640 |
| - 60-70 | -1.19 | 0.86 | -1.38 | 0.167 | -0.24 | 0.85 | -0.29 | 0.774 |
| - 70-80 | -2.26 | 0.87 | -2.59 | 0.010* | -0.05 | 0.88 | -0.06 | 0.956 |
| - >80 | -5.33 | 1.07 | -4.96 | <0.001* | -1.96 | 1.11 | -1.76 | 0.078 |
| NCCN risk group  (low risk as base case) |  |  |  |  |  |  |  |  |
| - Intermediate | -0.61 | 0.36 | -1.71 | 0.087 | 0.004 | 0.46 | 0.01 | 0.994 |
| - High | -2.49 | 0.43 | -5.86 | <0.001* | -0.55 | 0.54 | -1.03 | 0.304 |
| - Nodal | -5.12 | 1.00 | -5.12 | <0.001* | -2.10 | 1.07 | -1.97 | 0.049* |
| - Metastatic | -3.3 | 0.71 | -4.69 | <0.001* | -0.66 | 0.87 | -0.75 | 0.451 |
| - Not classifiable | 0.46 | 0.80 | 0.58 | 0.562 | 0.90 | 0.79 | 1.14 | 0.254 |
| Treatment Modality  (Active surveillance as base case) |  |  |  |  |  |  |  |  |
| - Prostatectomy | 0.55 | 0.33 | 1.66 | 0.098 | 0.65 | 0.44 | 1.48 | 0.140 |
| - Radiotherapy | -6.08 | 0.42 | -14.56 | <0.001* | -5.38 | 0.52 | -10.33 | <0.001* |
| - ADT | -3.16 | 0.67 | -4.69 | <0.001* | -1.63 | 0.87 | -1.88 | 0.060 |
| - Other | -0.74 | 1.28 | -0.58 | 0.563 | -0.53 | 1.30 | -0.41 | 0.683 |
| Remoteness classification  (Major cities as base case) |  |  |  |  |  |  |  |  |
| - Inner Regional | -1.54 | 0.33 | -4.65 | <0.001* | -0.59 | 0.35 | -1.71 | 0.087 |
| - Outer Regional | -1.10 | 0.57 | -1.92 | 0.055 | 0.27 | 0.59 | 0.47 | 0.641 |
| IRSAD in quarters  (top quarter as base case) |  |  |  |  |  |  |  |  |
| - 3^rd^ quarter | -0.73 | 0.37 | -1.98 | 0.048* | -0.52 | 0.36 | -1.44 | 0.149 |
| - 2^nd^ quarter | -1.37 | 0.37 | -3.67 | <0.001* | -0.73 | 0.39 | -1.89 | 0.059 |
| - Bottom quarter | -3.07 | 0.40 | -7.73 | <0.001* | -1.90 | 0.42 | -4.51 | <0.001* |

Supplementary Table 8. Univariate and multivariate linear regression, exploring contributions of disease characteristics, treatment modality, remoteness and socioeconomic status to scores in the EPIC-26 “Hormonal” domain. * indicates p < 0.05

|  | Univariate | | | | Multivariate | | | |
| --- | --- | --- | --- | --- | --- | --- | --- | --- |
|  | Estimate | SE | t | p | Estimate | SE | t | p |
| Age in years (<50 as base case) |  |  |  |  |  |  |  |  |
| - 50-60 | -2.37 | 1.19 | -1.99 | 0.046* | -0.96 | 1.13 | -0.85 | 0.394 |
| - 60-70 | -1.77 | 1.15 | -1.55 | 0.121 | 0.67 | 1.09 | 0.61 | 0.540 |
| - 70-80 | -2.16 | 1.17 | -1.85 | 0.064 | 2.74 | 1.13 | 2.42 | 0.015* |
| - >80 | -5.03 | 1.43 | -3.51 | <0.001* | 4.58 | 1.43 | 3.20 | 0.001* |
| NCCN risk group  (low risk as base case) |  |  |  |  |  |  |  |  |
| - Intermediate | -1.23 | 0.46 | -2.67 | 0.008* | -0.30 | 0.59 | -0.51 | 0.611 |
| - High | -7.39 | 0.55 | -13.47 | <0.001* | -5.14 | 0.69 | -7.44 | <0.001* |
| - Nodal | -13.38 | 1.29 | -10.37 | <0.001* | -8.21 | 1.37 | -5.98 | <0.001* |
| - Metastatic | -15.54 | 0.92 | -16.95 | <0.001* | -8.91 | 1.12 | -7.97 | <0.001* |
| - Not classifiable | 0.86 | 1.03 | 0.83 | 0.408 | 0.87 | 1.02 | 0.85 | 0.393 |
| Treatment Modality  (Active surveillance as base case) |  |  |  |  |  |  |  |  |
| - Prostatectomy | -1.79 | 0.44 | -4.10 | <0.001* | -0.43 | 0.57 | -0.74 | 0.455 |
| - Radiotherapy | -9.90 | 0.55 | -18.15 | <0.001* | -7.56 | 0.67 | -11.31 | <0.001* |
| - ADT | -16.08 | 0.88 | -18.31 | <0.001* | -9.94 | 1.11 | -8.93 | <0.001* |
| - Other | -2.17 | 1.67 | -1.30 | 0.195 | -0.90 | 1.67 | -0.54 | 0.591 |
| Remoteness classification  (Major cities as base case) |  |  |  |  |  |  |  |  |
| - Inner Regional | -2.41 | 0.44 | -5.48 | <0.001* | -0.61 | 0.45 | -1.36 | 0.173 |
| - Outer Regional | -1.99 | 0.76 | -2.60 | 0.009* | 0.77 | 0.76 | 1.03 | 0.305 |
| IRSAD in quarters  (top quarter as base case) |  |  |  |  |  |  |  |  |
| - 3^rd^ quarter | -1.69 | 0.48 | -3.50 | <0.001* | -1.28 | 0.47 | -2.74 | 0.006* |
| - 2^nd^ quarter | -3.27 | 0.49 | -6.61 | <0.001* | -2.24 | 0.50 | -4.51 | <0.001* |
| - Bottom quarter | -6.19 | 0.52 | -11.8 | <0.001* | -4.25 | 0.54 | -7.84 | <0.001* |
